# Respiration Signal Pattern Analysis for Doppler Radar Sensor with Passive Node and Its Application in Occupancy Sensing of a Stationary Subject

**DOI:** 10.3390/bios15050273

**Published:** 2025-04-27

**Authors:** Chenyan Song, Ehsan Yavari, Xiaomeng Gao, Victor M. Lubecke, Olga Boric-Lubecke

**Affiliations:** 1 Adnoviv, Inc., Honolulu, HI 96822, USA; song@adnoviv.com; 2 Aptiv, Inc., Carmel, IN 46032, USA; ehsan.yavari@aptiv.com; 3 Department of Electrical and Computer Engineering, University of Hawaii at Manoa, Honolulu, HI 96822, USA; gaoxiaom@hawaii.edu (X.G.); lubecke@hawaii.edu (V.M.L.)

**Keywords:** Doppler radar, occupancy sensor, cardiorespiratory motion, respiration signal

## Abstract

Doppler radar node occupancy sensors are promising for applications in smart buildings due to their simple circuits and price advantage compared to quadrature radar sensors. However, single-channel sensitivity limitations may result in low sensitivity and misinterpreted motion rates if the detected subject is at or close to “null” points. We designed and tested a novel method to eliminate such limits, demonstrating that passive nodes can be used to detect a sedentary person regardless of position. This method is based on characteristics of chest motion due to respiration, found via both simulations and experiments based on a sinusoidal model and a more realistic model of cardiorespiratory motion. In addition, respiratory rate variability is considered to distinguish a true human presence from a mechanical target. Sensor node data were collected simultaneously with an infrared camera system, which provided a respiration signal reference, to test the algorithm with 19 human subjects and a mechanical target. The results indicate that a human presence was detected with 100% accuracy and successfully differentiated from a mechanical target in a controlled environment. The developed method can greatly improve the occupancy detection accuracy of single-channel radar-based occupancy sensors and facilitate their adoption in smart building applications.

## 1. Introduction

With the global energy demand forecast to increase by 50% by 2050, compared to 2020 levels [1], there has been an increasing focus on energy efficiency and energy conservation. Building energy consumption accounts for about 40% energy consumption worldwide, and more than 50% of building energy is used for lighting and HVAC [2,3]. Research has shown that occupancy sensors can reduce lighting and HVAC energy use by as much as 80%, resulting in significant financial savings [4,5,6]. Besides providing a means of minimizing energy consumption, occupancy sensors have found a niche in the rapidly growing demand for “smart homes” and “smart buildings” with another growing trend in the technology of home and workplace automation [7,8]. Additional uses of occupancy sensors include security (by indicating that an area is occupied) and minimizing light pollution (by reducing the usage of lighting operating at night), whether it be outdoor lighting or indoor lighting emitting through windows or skylights.

An occupancy sensor is designed to detect the presence or absence of people in a space, usually in order to determine whether various electrically powered loads in that room (for example, lights and ventilation) should be turned on or not [9,10,11,12]. This is of particular advantage in hotels, commercial and government facilities such as office complexes, as well as communities such as military housing, college dorms, and public housing. When utilized properly, occupancy sensors can conserve a great deal of energy. This has led many businesses to purchase them voluntarily, and also resulted in laws and regulations mandating the use of occupancy sensors as an energy conservation measure [13].

The most common occupancy sensors use passive infrared (PIR), ultrasonic, and dual (combination of PIR and ultrasonic) technologies—each of which has its own advantages and disadvantages [14]. Regardless of which sensor type is selected, the goal is to gain the full advantages of occupancy sensor operation while avoiding possible negative outcomes, most notably erroneous switching. For example, a common issue with PIR sensors is that lights may be turned off while somebody is still in the room but has been motionless for several minutes. To avoid this issue, long time delay settings between the last detected motion and load switching are often used. However, even with delay settings of 20–30 min, false switching in occupied spaces still happens, provoking users to disable installed sensors, thus reducing energy savings and hampering further adoption [15]. Camera based occupancy sensors introduce a privacy concern, and blurred imaging systems that help preserve privacy introduce a significant computational burden [12].

**Figure 1:**
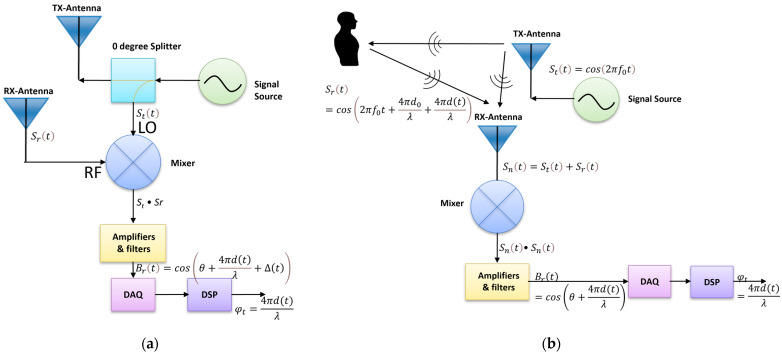
Block diagrams of classic single-channel radar sensor (**a**) and single-channel radar sensor utilizing a passive node as receiver (**b**). In both (**a**,**b**), *S_t_*(*t*) is the transmitted signal, *S_r_*(*t*) is the reflected signal, *B_r_*(*t*) is the baseband signal, *λ* is the wavelength of the transmitted signals, *d*(*t*) is the displacement of chest motion, and *φ_t_* is the demodulated phase shift proportional to *d*(*t*). (**a**) The transmitter and receiver are wired together. (**b**) The receiver does not have a physical connection with the transmitter. The passive node is placed in proximity with the transmitter. Therefore, the distance between the subject and RX antenna is approximated as the distance between the subject and the TX antenna and the distance between the TX and RX antenna can be ignored when deriving the mathematical expression of *B_r_*(*t*).

Doppler radar occupancy sensing is an emerging technology that has potential to eliminate false switching by detecting a true human presence based on measurements of human respiration, while preserving privacy [16,17,18,19]. These sensors were demonstrated effective for occupancy sensing with over 90% accuracy, with successful respiratory and heart rate detection for occupants in natural positions and orientations throughout a 3.4 × 8.5 m^2^ conference room, and HVAC energy savings of over 20% [20,21]. In addition, the advent of integrated system on chip (SoC) low-power microprocessor/RF-transceivers provides a new platform for a combination of sensing, processing and communication, which can form the core of a WSSN for applications such as “smart building” systems. Traditional occupancy sensors are usually hard wired with loads that they control, while also getting access to power lines. This creates a barrier to the adoption of new technologies, which may only be available during renovation or new construction. Wireless sensors that communicate with loads through a communication link and are powered with batteries or photovoltaic cells can be installed without access to electric wiring, and thus significantly reduce the complexity and cost of installation [6]. A wireless occupancy sensor based on Doppler radar (Figure 1b) was proposed in [22]. This sensor architecture can leverage communication signals existing in the environment of interest. Unlike CSI based physiological sensing [23,24] that requires access to communications hardware to obtain CSI, the method proposed in [22] simply uses a communication signal as a signal source. A custom built, low-cost, low-power compact receiver (sensor node) combines a communication signal with signals reflected from occupants in the environment and discerns physiological signals from the mixture of those two signals. This technique was demonstrated to be effective with various communication signals [22]. The sensor node configuration is also suitable for energy harvesting, thus potentially extending the battery life, or even providing a self-powered occupancy sensor solution.

This paper is focused on SoC-based Doppler radar occupancy sensors that can be used to detect and report the true presence and activity of humans by adding sensor nodes to off-the-shelf low-power SoC platforms. These occupancy sensors integrate the capabilities of sensing, processing and communications in a single device, thus providing the key to small, low-cost, and low-power wireless sensors for “smart buildings”. A novel algorithm was proposed to ensure detection accuracy regardless of occupant position. The effectiveness of this technique was demonstrated in a proof-of-concept study using a mechanical target simulating a human presence, and demonstrated a high degree of accuracy in human testing. Section 2 describes the sensor hardware configuration, human signal modeling, and experimental testing set-up and procedure. The most challenging scenario for occupancy sensors is a single, sedentary occupant, and thus the testing was carried out in that configuration. Section 3 describes the results of extensive sensor testing with a mechanical target and 19 human subjects, and presents an algorithm design for true presence detection. Section 4 discusses the main accomplishments highlighted in this paper.

## 2. Materials and Methods

### 2.1. Doppler Radar Based Sensor with Passive Sensor Node

According to Doppler theory, signal reflected from an object with periodic movement, such as chest motion due to cardiorespiratory activities, contains the same frequency as the transmitted signal, but with a time-varying phase shift that is proportional to the displacement of the movement over time. This motion-modulated phase shift and hence motion related information, such as displacement, moving rate or frequency, can be measured and characterized by a radar system, such as a simple single-channel Doppler radar. Although quadrature radar shows improved sensitivity and signal fidelity compared to single-channel radar sensors, it has a higher cost, more power consumption and greater complexity due to its dual-channel architecture and higher signal processing workload. Therefore, a single-channel radar sensor was adopted for the purpose of presence detection in this research.

Figure 1a shows a block diagram of a single-channel Doppler radar transceiver. In the transmitter, a continuous wave radio signal is generated by the RF signal source. This RF signal is split in two with a power splitter so part of it is transmitted by an antenna, and the other part is fed to an LO input port of the mixer in the receiver. In the receiver, the reflected signal received by the other antenna enters the mixer’s RF input port. The mixer will downconvert the signal to baseband, which contains the motion information. This baseband signal is then amplified and filtered before it is digitized to produce the phase shift proportional to displacement. The transmitted and reflected signals in this process can be represented by the following equations, respectively:

(1) $$S_{t}\left(t\right)=\cos\left(2\pi f_{0}t+\phi \left(t\right)\right),$$

(2) $$S_{r}\left(t\right)=\cos\left(2\pi f_{0}t+\frac{4\pi d_{0}}{\lambda }+\frac{4\pi d(t)}{\lambda }+\phi \left(t-\frac{2d_{0}}{c}\right)\right),$$

where *f*_0_ is the frequency of the transmitted signal, *t* is the elapsed time, *ϕ*(*t*) is the phase noise of the oscillator in the transmitter, *d*_0_ is the nominal distance between the subject and the radar sensor, and *d*(*t*) is the displacement due to respiration and heartbeat. The baseband signal is obtained by low-pass filtering the product of the transmitted and reflected signals via a mixer, as shown below:

(3) $$B_{r}\left(t\right)=\cos\left(\theta +\frac{4\pi d(t)}{\lambda }+∆\phi (t)\right),$$

where ∆ϕ(t) is the residual phase noise and θ is the phase shift related to the nominal distance *d*_0_ to the target with a fixed phase compensation $\theta_{0}$ for a given radar system. θ can be expressed as:

(4) $$\theta =2\pi f_{0}t+\frac{4\pi d_{0}}{\lambda }+\theta_{0}.$$

Equations (3) and (4) illustrate that the base band signal, and hence respiratory signal, varies with respect to the nominal distance *d*_0_ for a chest motion *d*(*t*): when θ is an odd multiple of *π*/2, i.e., *d*_0_ is an odd multiple of an eighth of the wavelength (*λ*/8) of the transmitted signal, the subject is at “optimum” points, where the based band signal is linearly proportional to the displacement, as shown below:

(5) $$B_{r}\left(t\right)\approx \frac{4\pi d\left(t\right)}{\lambda }+∆\phi ;$$

and when θ is an integer multiple of π, i.e., *d*_0_ is an integer multiple of a quarter of the wavelength (*λ*/4) of the transmitted signal, the subject is at ”null” points, where the based band signal is proportional to the square of the time-varying displacement *d*(*t*) and becomes less sensitive to physiological motion:

(6) $$B_{r}\left(t\right)\approx 1-{\left(\frac{4\pi d(t)}{\lambda }+∆\phi \right)}^{2};$$

Equation (4) illustrates that where the null and optimum points are depends on the distance between the radar and the subject, and that the distance between these two points next to each other is only an eighth of a wavelength apart. At a frequency of 2.4 GHz, the “null” and “optimum” points will occur every 1.5 cm. Therefore, optimal sensitivity will be hard to maintain due to variations in the positions of the radar transceiver and the subject. To avoid this problem, we designed and tested a method with a single-channel Doppler-radar based occupancy sensor with a passive node that can detect a sedentary person regardless of where the person is in a room. This method is based on the characteristics of chest motion due to respiration, and it has been tested via both a simulation and an experiment with a simplistic sinusoidal model and a more complex model for cardiorespiratory motion.

This paper employs an unconventional single-channel Doppler-radar system built with a commercially available transmitter and an add-on passive node as the occupancy sensor. Different from a classic single-channel radar sensor in which the transmitter and receiver are wired together (Figure 1a), the sensor in this paper uses a passive sensor node as the receiver, which makes use of the ambient RF signals generated by an existing electronic device, the commercially available transmitter in this paper, and hence does not have a physical connection with the transmitter. Figure 1b shows the block diagram and operation concept of such a radar system with a sensor node consisting of an antenna and a mixer. The passive sensor node receives both the direct signal from the transmitter and a motion-modulated phase shift signal reflected from moving objects in the room. The baseband output can be produced by splitting the received signal into two parts and passing each part through a mixer via its LO and RF ports, respectively. The output signal in a form similar to Equation (3) is shown in Figure 1b, demonstrating the radar sensor with a passive node utilizing air-coupling is essentially a single-channel radar.

**Figure 2:**
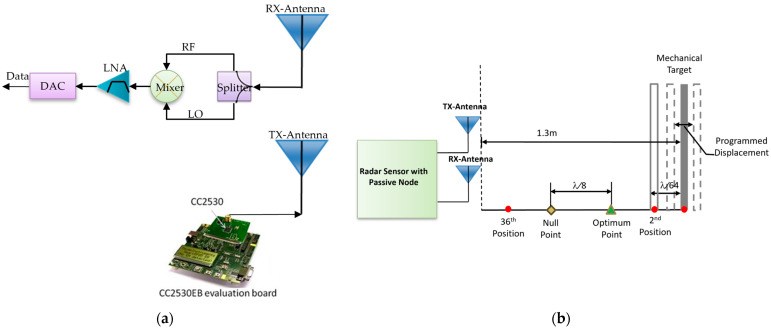
(**a**) Block diagram and operation demonstration of the passive node radar sensor. (**b**) Illustration of the experimental setup to study the impact of the position of a periodic moving target on the demodulated respiratory signal. The mechanical target makes an oscillation programmed with sinusoidal models and complex models expressed by Equations (9) and (10) to emulate human cardiorespiratory motion. The wavelength *λ* in correspondence with the sensor transmitting frequency at 2.045 GHz, one of the CC2530 SoC evaluation board’s operational frequencies, is 0.125 m. Each new position of the target is closer to the sensor by *λ*/64 than the previous position.

Figure 2a illustrates how the radar sensor with a passive node was constructed for this paper. The transmitter, composed of a CC2530 SoC evaluation board [25] purchased from Texas Instruments (Dallas, TX, USA) and one ASPPT2988 antenna with 8 dBi gain and a 60-degree E-plane beamwidth purchased from Antenna Specialist (Bloomingdale, IL, USA), generates a continuous RF signal. The passive sensor node, consisting of a 3 dB ZFSC-2-2500 power splitter bought from Mini-Circuits (Brooklyn, NY, USA), a Mini-Circuits ZFM4212 mixer and the other ASPPT2988 antenna, serves as the RF front end of the receiver. The two antennae are located close to each other to provide a strong coupling signal for the LO port of the mixer. The baseband signal is fed through a Stanford Research System (Sunnyvale, CA, USA) Model SR560 LNA for amplification and filtering. The output of LNA is digitized and recorded by a National Instruments (Austin, TX, USA) USB-6259 DAC for further processing via a PC. This paper reports the research that was conducted on the most challenging application of this sensor, i.e., detecting the room occupancy by a single, sedentary person.

### 2.2. Methods

Since the sensor with add-on passive node employed in this research is a single channel Doppler radar sensor, its sensitivity will fluctuate and hence impacts its detection accuracy depending on where the target is in respect to the sensor [26]. To find a method that overcomes the disadvantage of inconsistent sensitivity and hence improves its overall accuracy, we first studied the pattern of the variation in sensitivity vs. the position change by performing simulations. Simulations were performed using MATLAB R2021b in which mathematic equations were employed to calculate the displacement of the cardiorespiratory motion over time for each position under test. Next, experiments were conducted to verify the simulation results, using a mechanical target that emulates chest movement due to cardiorespiratory effort. Based on the respiratory signal pattern found, a detection algorithm was then developed to determine the presence of a stationary person in the room. Finally, human tests were run to test the validity of the algorithm.

#### 2.2.1. Respiration Signal Pattern Analysis with Sinusoidal Models

Two sinusoidal signals, *Asin*2*πf*_1_*t* and *Bsin*2*πf*_2_*t*, were assumed to present displacements of respiration and heartbeat, respectively, in Equation (3). Here, *f*_1_ is the frequency of respiration, *f*_2_ the heartbeat frequency, *A* and *B* are the corresponding amplitude, *f*_1_ < *f*_2_, and *A* >> *B*. Through mathematical derivation, the baseband signal for a subject at “optimum” and “null” points can be expressed below, individually:

(7) $$B_{r}\left(t\right)\approx Asin2\pi f_{1}t+Bsin2\pi f_{2}t+∆\phi (t),$$

(8) $$B_{r}\left(t\right)\approx 1-\frac{1}{2}[\left(A^{2}+B^{2}\right)-{A^{2}\cos\left(4\pi f_{1}t\right)-B}^{2}\cos\left(4\pi f_{2}t\right)-2AB\cos2\pi {(f}_{1}+f_{2})t+2AB\cos2\pi {(f}_{1}-f_{2})t].$$

In addition to confirming the findings from Equation (6), the expression above also suggests that a frequency obtained in this way may no longer reflect the real rate of the subject’s movement. Depending on the magnitudes of *A* and *B* and the bandwidth of the filter, the baseband output could include one or more of the following: the doubled original frequencies, the sum of the respiration and heartbeat frequencies, and the difference of them. Since the amplitude of respiration *A* is usually much greater than that of the heartbeat, doubling of the real respiration frequency is expected to dominate in the spectrum after filtering is applied.

When a subject is at a position between null and optimum points, the phenomenon will be too complicated to describe with a simple mathematical expression. Therefore, a MATLAB simulation based on Equation (3) and a sinusoidal model was performed to study how the radar sensor output changes with the varied positions of the subject in respect to the radar with the critical parameters set as: respiration frequency *f*_1_ = 0.25 Hz; heartbeat frequency *f*_2_ = 1.25 Hz; magnitudes *A* = 1, *B* = 0.03; carrier frequency *f*_0_ = 2.045 GHz in line with the operation frequency of the CC2530 SoC evaluation board; and the nominal distance *d*_0_ set at 1 m first and then decreased with 64th of the radar carrier wavelength (*λ*/64) each time for a total of 36 simulation runs.

To verify the validity of the mathematical simulation, a mechanical target programmed to make the two sinusoidal motions above was tested by a Doppler radar sensor as shown in Figure 2a. The CC2530 evaluation board was programmed to transmit a 2.405 GHz CW signal at 4.5 dBm output power. The target was programmed to oscillate with a combination of two sinusoidal waveforms at the frequencies of 1.25 Hz and 0.25 Hz and the maximum displacements of 1 cm and 0.03 cm, respectively, which emulate the cardiorespiratory chest movement. The mechanical target was placed about 1.3 m away from the radar sensor originally before it started the programmed oscillation. In accordance with the variation of nominal distance *d*_0_ in the simulation, the mechanical target was brought closer to the sensor each time by *λ*/64 at 2.405 GHz to make the sinusoidal oscillations. The same measurement was repeated 36 times for each starting point. Figure 2b shows an illustration of the experimental setup.

In all testing, the baseband signal outputs from the sensor node were amplified by a factor of 200 and were subjected to 6 dB/octave low pass filtering with a cutoff frequency of 30 Hz by the LNA. Finally, the signals were digitized and recorded by the USB-6259 DAC with a sampling rate of 100 Hz. The experimental data were stored on a PC and post-processed using MATLAB to find the pattern of the demodulated signal vs. the initial positions of the target.

#### 2.2.2. Respiration Signal Pattern Analysis with Complex Signal Model

In reality, human respiration and heartbeat are not in the form of a sinusoidal wave. Therefore, more realistic models were adopted for the respiration and heart signals, respectively, as follows [20]:

(9) pRt=sinπ   pfRt,

(10) $$p_{H}\left(t\right)=e^{t/\tau }+\left[\left(\frac{\sqrt{2}}{\omega_{0}\tau }-1\right)\sin\frac{\omega_{0}t}{\sqrt{2}}-\cos\frac{\omega_{0}t}{\sqrt{2}}\right]e^{-\omega_{0}\tau /\sqrt{2}}.$$

where the chest motion due to respiration is modeled with a sinusoidal half-cycle with a rounded cusp, $f_{R}$ is the respiratory frequency and *p* controls the rounding of the cusp and the general shape of the signal, while the heart signal is modeled with an analog pulse of an exponential *e^t/τ^*, with time constant *τ*, filtered by a critically damped second-order Butterworth filter with cutoff frequency ω_0_. The pulse shape for the heart signal repeats at 1/*f_H_* with a heartbeat frequency *f_H_*.

Using Equations (9) and (10) to replace the two sinusoidal signals in the previous section, a MATLAB simulation closer to the real situation was performed by repeating the same procedures described in the previous section, with the value listed for the following parameters: the peak-to-peak amplitude *A* of the respiration signal, 1, and *B* for the heartbeat, 0.03; the heart rate *f_H_*, 1.25 Hz; and the respiration rate *f_R_*, 0.25 Hz. The other parameters, *τ* = 0.05, *ω*_0_ = 2π, and *p* = 3, were chosen to best represent typical real data.

In the experiment to examine how the subject location impacts the demodulated output signal, the same setup and process described in the previous section were employed, except the mechanical target was programmed to emulate the cardiorespiratory chest motion using Equations (9) and (10) with the parameters set as above.

#### 2.2.3. Algorithm to Determine the Occupancy of a Stationary Subject

An algorithm was developed first to determine the presence of a stationary person in a room based on the spectrum characteristics of a demodulated respiration signal found via the research described in the previous sections and the nature of a time-varied respiration rate [20]. This algorithm focused on the occupancy detection of a stationary subject since large amplitude signals produced by locomotion and fidgeting are easily discerned by Doppler radar as well as other commercially available occupancy sensors.

#### 2.2.4. Occupancy Determination of a Stationary Subject

The proof-of-concept tests were run with a stationary person sitting in front of the radar occupancy sensor at a distance of 1.2 m, using the same experimental setup as in Section 2.2.1 and Section 2.2.2. A total of 19 subjects were tested by following the University of Hawaii approved human testing protocol CHS 14884.

The data recorded via the USB-6259 DAC were processed by a MATLAB code written in the form of the occupancy algorithm developed above. Since the algorithm is based on the characteristics of respiratory motion in the spectrum domain, the respiratory frequency for each subject was extracted from 1 min data after FIR low pass filtering and FFT.

In addition, to extract the inherent variability in the respiration rate, a short time Fourier transform was adopted in which the collected 1 min data were divided into six 10 s windows, and then FFT was applied to the windowed data to find the peak in the spectrum domain for each window. As a comparison, the data collected using a mechanical target programmed to oscillate at a constant rate of 0.25 Hz under the same test setup were processed using same windowed FFT to obtain the motion frequency for each 10 s window. By doing this, an object making a periodic motion with constant rate can be differentiated from a sedentary person breathing with a rate changing over time.

In parallel with the Doppler radar sensor test setup, an infrared camera system monitoring the movement of the chest activity was running to take the same measurement for reference. ARTTRACK2 infrared (IR) cameras manufactured by Advanced Realtime Tracking GmbH & Co. KG (Weilheim in Oberbayern, Bavaria, Germany) with a DTrack central PC with Sync-card and proprietary processing software were used. Two infrared cameras formed a stereo vision, enabling the tracking of three-dimensional coordinates of markers within their field of view. The y-axis, which is horizontal and perpendicular to the subject’s frontal plane, was used to calculate the average displacement from the marker data, corresponding to the radar measurement.

## 3. Results

### 3.1. Respiration Signal Pattern Analysis Results

#### 3.1.1. Results Obtained Using Sinusoidal Models

A MATLAB code was written to plot the simulated waveform of the baseband signal based on the sinusoidal models and the FFT of the baseband signal for each nominal distance preset in the simulation as described in Section 2.2.1. A total of 36 sets of results, which are presented in the time domain and spectrum domain, were obtained for the programmed 36 positions.

**Figure 3:**
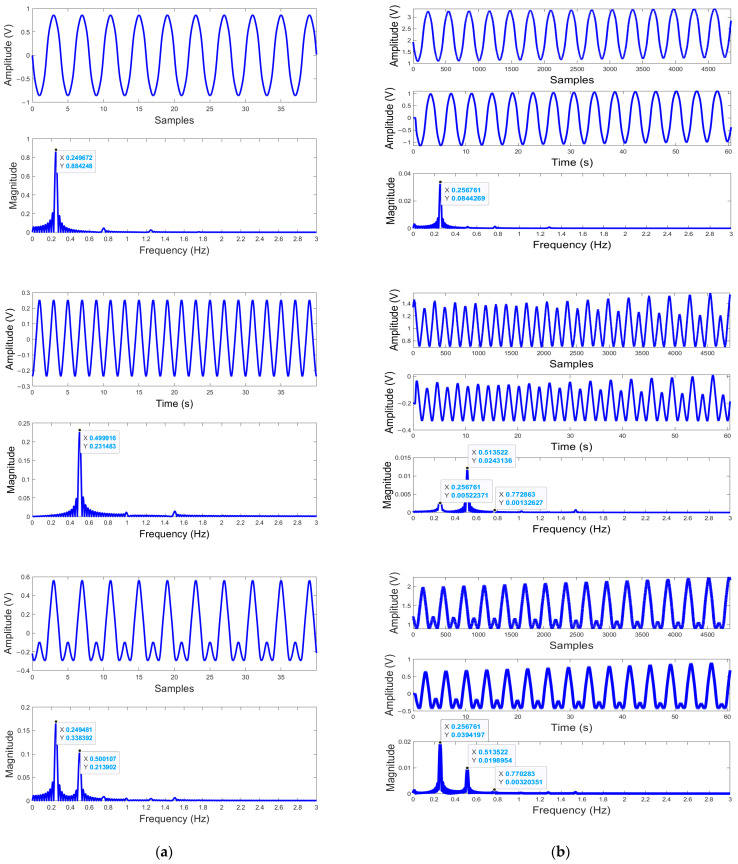
Example simulated and experimental results using a sinusoidal model, both shown in the time domain and frequency domain for “optimum”, “null” and “in-between” points from top to bottom. (**a**) Simulated results: For each example point, the calculated baseband signal is plotted in the time domain on top, and two respiration frequencies can be identified in the bottom plot—0.25 Hz and/or 0.5 Hz, by which the peak(s) become dominant in the spectrum domain after the time-varied signals go through FFT; (**b**) Experimental results. For each example point, the raw data are plotted at the top and the DC-cancelled and filtered data are shown in the middle, both in the time domain, and the spectrum data after FFT are shown at the bottom, where the peak of 0.2568 Hz and/or the peak of 0.5136 Hz are identified.

Figure 3a shows three examples of these results, which correspond to three representative positions: “optimum” point, “null” point, and “in-between” from top to bottom. In the top example, an obvious peak presents at 0.25 Hz in the graph of the spectrum domain. This means the target is at a “optimum” point, the respiratory frequency set for the simulation. The middle example indicates that the target is at a “null” point, since the dominant peak shows up at 0.5 Hz, double the real input for the respiration frequency. Comparing the maximum amplitudes of the time-varied signal waveforms in both examples, the one in the middle example is much less than the one in the top example. In addition, the difference of the nominal distances set for the two examples is *λ*/8. These observations confirm that the example results obtained are at “optimum” and “null” points, respectively. The bottom example shows both peaks of 0.25 Hz and 0.5 Hz with different magnitudes in the spectrum. This result is obtained for the nominal distance of a position between the “null” and “optimum” points since the “null” and “optimum” points repeat every *λ*/4 and they are *λ*/8 apart from each other.

Figure 3 also contains the example results obtained from the experiment in which a mechanical target was programmed to emulate human cardiorespiratory movement as described in Section 2.2.1. Figure 3b shows the measured real oscillation frequency, 0.2568 Hz and its second harmonic, which are very close to the programmed real motion frequency, 0.25 Hz and its second harmonic. Depending on where the target initially was relative to the “null” or “optimum” points, i.e., the nominal distance between the target and the radar sensor, the appearance and the strength of these two signals vary as demonstrated by the simulation.

**Figure 4:**
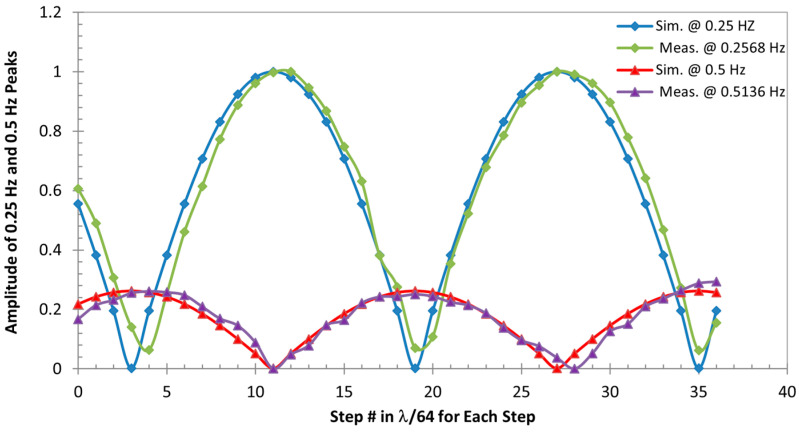
Illustration of demodulated sensor output for the sinusoidal model–real respiratory signal (0.25 Hz for the simulation and 0.2568 Hz for the experiment) and misinterpreted signal (0.5 Hz for the simulation and 0.5136 Hz for the experiment) changes with the position of the target relative to the sensor, which is expressed by the incremental # of *λ*/64, where *λ* = 0.125 m.

Figure 4 summarizes the results obtained for all 36 positions, showing how the magnitude of the demodulated respiration signals varies with different initial positions of the target with respect to the radar sensor, i.e., the change in the nominal distance between the target and the sensor. For all positions simulated, one or both of two frequencies were demodulated, 0.25 Hz—the real respiratory frequency and 0.5 Hz—the second hormonic of the real one. As shown in the plot, when the magnitude of the 0.25 Hz peak reaches the maximum at the “optimum” points, the magnitude of its second harmonic is at a minimum, or no obvious 0.5 Hz peak is found at “null” points, and vice versa. It is also found that as the input nominal distance comes closer to that of the first “null” point, or the target moves closer to it, the magnitude of the real respiratory signal becomes smaller and less than that of its second harmonic after passing a certain point where both signals are equally strong. Corresponding to this, the change in the 0.5 Hz signal has the opposite trend. When the target continues to move closer to the first “optimum” point, the magnitude of 0.25 Hz becomes bigger while its second harmonic becomes weaker. The real respiratory signal dominates its second harmonic after passing a certain point where both signals show equal strength. Once the target passes the first “optimum” point and moves to the second “null” point, the same pattern repeats. From Figure 4, we can also tell that each consecutive “optimum” and “null” points are *λ*/8 apart, and each “optimum” and “null” points repeatedly appear every *λ*/4.

To compare with the simulation, the magnitude of respiratory frequencies, including the real respiratory frequency and its second harmonic, obtained from experiment at each of the 36 positions where the target starts the cardiorespiratory movement modeled by sinusoidal signals are also plotted in Figure 4. The comparison shows that the appearance and strength of the real motion signal and its second harmonic change with the position of the target, closely following the simulation results.

#### 3.1.2. Results Obtained Using Complex Signal Models

Similar to using a sinusoidal model, MATLAB simulations and experiments with mathematical models more realistically presenting respiration and heartbeat signals were implemented for 36 positions. The results were recorded and processed in both the time domain, showing the amplitude change of the demodulated respiratory signal over time, and in the frequency domain, showing the strength and frequencies of this signal.

**Figure 5:**
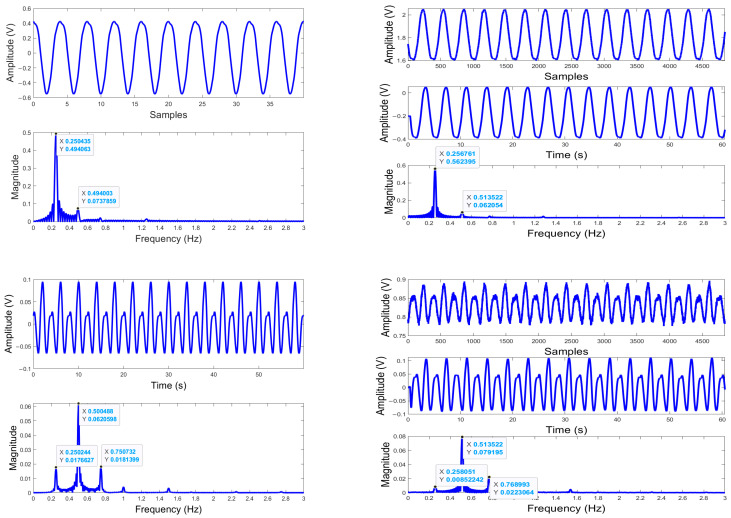

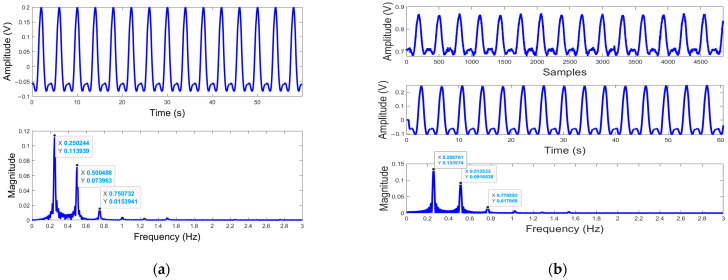
Examples of results in the time domain and frequency domain for “optimum”, “null” and “in-between” points from top to bottom, using a more complex but realistic model. (**a**) Simulated results: For each example point, the calculated baseband signal is plotted in the time domain on top, and two respiration frequencies can be identified in the bottom plot—0.25 Hz and/or 0.5 Hz, by which the peak(s) become dominant in the spectrum domain after the time-varied signals go through FFT; (**b**) Experimental results. For each example point, the raw data are plotted at the top and the DC-cancelled and filtered data are shown in the middle, both in the time domain, and the spectrum data after FFT are shown at the bottom, where the 0.2568 Hz peak and 0.5136 Hz peak are identified.

**Figure 6:**
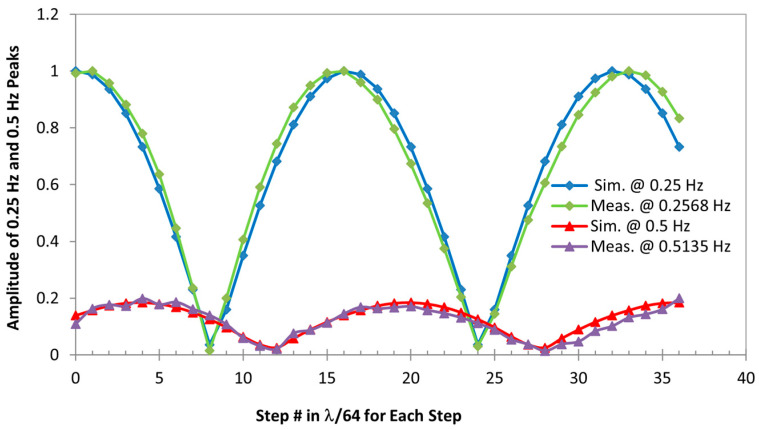
Illustration of demodulated sensor output for a complex but more realistic model–how the real respiratory signal (0.25 Hz for the simulation and 0.2568 Hz for the experiment) and a misinterpreted signal (0.5 Hz for the simulation and 0.5136 Hz for the experiment) changed with the position of the target relative to the sensor, which is expressed by the incremental # of *λ*/64, where *λ* = 0.125 m.

After analyzing all 36 results obtained using more realistic models, we found that where the peaks of the real respiratory frequency and its harmonics appear in the spectrum and how the strength of the real and misinterpreted frequencies vary with the nominal distance (or the positions of the target in respect to the radar sensor) follow a similar pattern as what was found with sinusoidal models. That is: at “optimum” points, peaks at the real respiratory frequency—0.25 Hz for the simulation and 0.2568 Hz for the experiment—present with a much larger magnitude; at “null” points, peaks at the second harmonic of the real one—0.5 Hz for the simulation and 0.5136 Hz for the experiment—dominate; and between the “optimum” and “null” points, whether peaks of the real one or its second harmonics dominate depends on how far or close the point is relative to the “optimum” or “null” points, i.e., if the target is at the points closer to the “optimum” than the “null” points, the real signal peak has a bigger magnitude and vice versa if the target is closer to the “null” points than the “optimum” points. One notable difference compared to the sinusoidal signals is that the second harmonic “null” is offset from the fundamental “optimum” and vice versa. This is due to the model itself, which has relatively strong harmonics [27]. The position of the second harmonic peak is affected by both the target position and the harmonic context of the signal itself. This pattern is illustrated by Figure 5 and Figure 6.

### 3.2. Occpancy Determination of a Stationary Subject

#### 3.2.1. Algorithm Developed to Determine the Occupancy of a Stationary Person

**Figure 7:**
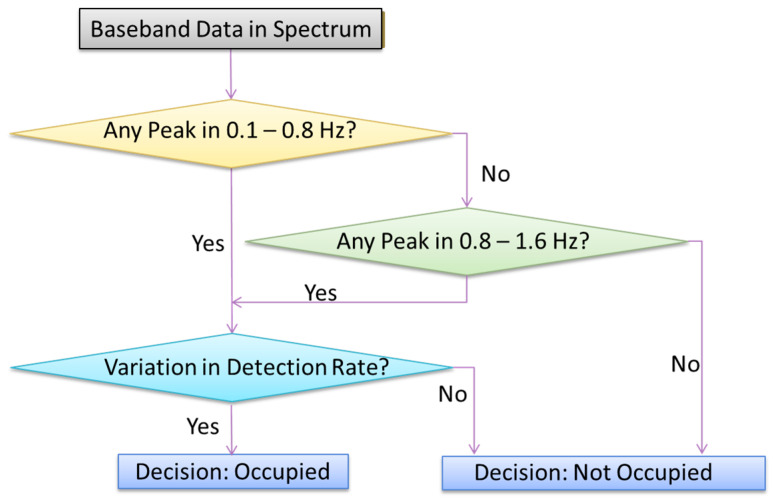
Flow chart of the algorithm to detect the occupancy of a room by a sedentary person.

The results from the simulation and experiment using sinusoidal and more complex models indicate that the radar-based occupancy sensor built with a passive node can detect two signals: the real respiration signal or its second harmonic. Whether one or both of them will show up in a spectrum depends on the position of the subject relative to the “optimum” or “null” points. Therefore, by checking if there is a peak at the respiration frequency or a peak at its second harmonic, or both peaks appearing in the spectrum, we can determine the presence of a stationary person in a room. Since the human respiration frequency range is usually within 0.1–0.8 Hz, we can search for peaks between 0.1–1.6 Hz as our occupancy detection baseline. To improve the efficiency, we can scan the frequency between 0.1–0.8 Hz first; if no peaks are found, then we expand the searching scope to 0.8–1.6 Hz. In addition, to rule out what is identified in the spectrum range of 0.1–1.6 Hz is not a mechanical target, such as fans or AC, moving with a constant rate, the motion rate demodulated by the sensor will be examined over time. The overall flow chart of the proposed algorithm is presented in Figure 7.

#### 3.2.2. Proof-of-Concept Test Results Using the Occupancy Algorithm for Detecting a Stationary Person

In the proof-of-concept test described in Section 2.2.4, the respiration frequency and its harmonics for each of the 19 subjects in the test were first measured using the proposed radar system with a passive node. The testing results were checked by the IR cameras.

**Figure 8:**
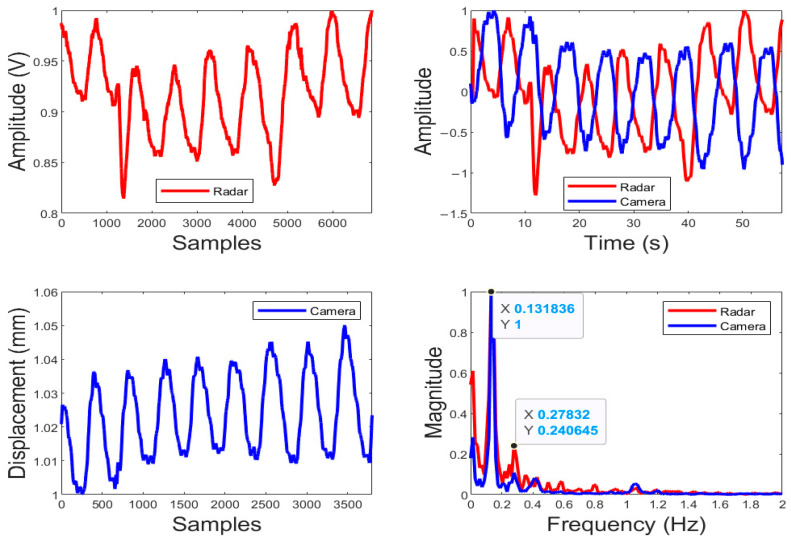
Detection of Subject #16 by the radar occupancy sensor with a passive node. The top left trace is the occupancy sensor raw data, the bottom left trace is the IR camera raw data, the top right traces are the filtered data for both the sensor (red) and reference camera (blue) in the time domain, and the bottom right traces show the frequency spectrum after FFT for both the radar sensor (red) and reference camera (blue). For the purpose of comparison on the same baseline, the data shown in the right top plot were normalized to the maximum output of itself for the sensor and camera, respectively.

As an example, Figure 8 shows the demodulated respirational signal in the time domain and frequency domain for subject #16. The measurement was taken by the radar occupancy sensor and an IR camera as a reference. Applying the occupancy algorithm developed in Figure 7, two peaks—a dominant one at 0.13 Hz and its second harmonic at 0.27 Hz—falling in the primary searching range of 0.1–0.8 Hz were identified in the spectrum by both the radar sensor and the camera. Although the radar data do not follow the reference data in the time domain exactly, due to radar capturing a signal from a larger area, the frequency domain results match with each other very well. Since the radar detects the movement in a broader area than the camera, it is reasonable that there is a phase discrepancy between the radar and reference camera as shown in the time domain.

Table 1 summarizes the radar testing results and the IR camera ground-checking results for all 19 subjects. The results in Table 1 clearly indicate that not only are the detected dominant respiration frequencies measured by radar very close or equal to those measured by the IR cameras, but also, they fall into the range of 0.1–0.8 Hz for all subjects. Some of the dominant peaks are accompanied by second and even third harmonics due to the location of the subject and possibly the strong harmonics of the chest movement itself. Following the flowchart of the proposed algorithm in Figure 7, we can make a preliminary judgment that a human presence was detected for every one of the 19 subjects.

According to the algorithm, the next step is to exclude the interference of the periodic mechanical movement by checking if a time-varying respiration feature exists for each subject’s data taken via the radar sensor system presented in this research. Table 2 displays the extracted dominant frequency, i.e., the radar-measured respiration frequency for each 10 s sub-window for the whole 1 minute data taken for each subject. It illustrates that the measured respiration frequency is not a constant across the whole 1 min recording time. This confirms the preliminary judgment is correct in accordance with the algorithm. Table 2 only contains the radar test results, since the accuracy of the data has been validated by IR cameras as shown in Table 1.

As a comparison, the testing results on the mechanical target (#20) are also included in Table 2. When a mechanical target like #20 appears, the algorithm will first determine there is a person in the room since the radar detects a sedentary person with respiration frequency at 0.29 Hz. The target was programmed purposely to oscillate at 0.29 Hz, in the range of the preset respiratory frequencies. However, the designed algorithm will eventually exclude the possibility of a false positive alarm that can be triggered by the presence of the mechanical target due to its constant moving rate over time, as shown in Table 2.

Using the algorithm designed in accordance with the respiration pattern in the spectrum, we successfully detected all 19 sedentary subjects in a laboratory setup and excluded the possible misjudgment on a mechanical target with a constant moving rate.

## 4. Discussion

This paper presented a theoretical background, simulation results, and experimental validation of a Doppler radar node occupancy sensor concept. This is the first comprehensive study using sensor node architecture for occupancy sensing. An algorithm was developed to overcome the sensor node single-channel configuration sensitivity limitation, and it was demonstrated effective with a mechanical target and 19 human subjects. MATLAB simulations were carried out using sinusoidal and more complex models of combined respiration and heartbeat chest motion, and a mechanical target was programmed to emulate both models. Close agreement between the simulated and experimental results provided repeatable patterns between the fundamental and second harmonic amplitudes of the simulated respiration signals that formed the basis of the algorithm development. With this spectrum of characteristics in mind, searching for the peaks in the typical respiration frequency range of 0.1–0.8 Hz and double that is the baseline of the occupancy detection algorithm, as well as looking for variations in the rate within this range. Breath to breath variations are expected for both normal and abnormal breathing patterns [28]. Human testing with 19 subjects proved the validity of the occupancy detection algorithm with 100% accuracy. Future research directions include leveraging existing communication signals instead of a dedicated transmitter for occupancy sensing, and self-powering sensor nodes through energy harvesting [29]. While the presence of other RF sources in the environment may saturate the receiver output, it is more likely that multiple sources would provide spatial diversity that may be leveraged to enhance signal quality.

## 5. Patents

Patent No. US-10620307-B2 contains the results from the work reported in this manuscript.

## Figures and Tables

**Table 1:** Respiration frequencies of 19 subjects in the test measured by the radar sensor and IR cameras.

Subject	f0 ^1^ by Radar	f1 ^1^ by Radar	f2 ^1^ by Radar	F ^1,2^ by IR Camera
1	0.23 ^3^	0.46	-	0.23
2	0.19	0.39 ^3^	-	0.36
3	0.27	-	-	0.27
4	0.26	-	-	0.28
5	0.16	0.32 ^3^	-	0.33
6	0.34	-	-	0.34
7	0.24 ^3^	0.49	-	0.24
8	0.24		-	0.23
9	0.14 ^3^	0.28	0.42	0.14
10	0.21	-	-	0.21
11	0.15	-	-	0.15
12	0.26	-	-	0.26
13	0.28 ^3^	0.56	-	0.28
14	0.17 ^3^	0.35	-	0.17
15	0.27	-	-	0.27
16	0.13 ^3^	0.28	-	0.14
17	0.21 ^3^	0.42	0.64	0.21
18	0.32	-	-	0.33
19	0.26	-	-	0.26

^1^ The frequencies were obtained via FFT on 1 min data. ^2^ The fundamental respiration frequency measured by ARTTRACK2 IR cameras for each subject. This result was used to check the validation of the result measured by the radar system. ^3^ When there are multiple peaks in the spectrum domain, where the highest peak appears is considered as the radar measured fundamental respiration frequency.

**Table 2:** Detected dominant respiration rate vs. time for subjects #1–#19 and the mechanical target (#20).

Subject	Window 1 ^1^	Window 2 ^1^	Window 3 ^1^	Window 4 ^1^	Window 5 ^1^	Window 6 ^1^
1	0.18	0.27	0.27	0.18	0.27	0.18
2	0.10	0.39	0.10	0.39	0.39	0.29
3	0.29	0.29	0.29	0.29	0.23	0.23
4	0.12	0.23	0.06	0.12	0.41	0.12
5	0.29	0.29	0.35	0.35	0.12	0.06
6	0.41	0.35	0.35	0.29	0.29	0.35
7	0.29	0.23	0.18	0.23	0.23	0.23
8	0.12	0.23	0.12	0.23	0.06	0.06
9	0.12	0.35	0.12	0.29	0.12	0.06
10	0.18	0.18	0.23	0.18	0.23	0.23
11	0.18	0.12	0.12	0.18	0.18	0.18
12	0.23	0.29	0.12	0.23	0.06	0.12
13	0.26	0.26	0.29	0.29	0.26	0.26
14	0.23	0.29	0.23	0.18	0.12	0.12
15	0.29	0.29	0.29	0.29	0.29	0.35
16	0.12	0.18	0.12	0.12	0.12	0.12
17	0.23	0.23	0.18	0.23	0.18	0.18
18	0.12	0.12	0.35	0.41	0.29	0.35
19	0.23	0.23	0.29	0.29	0.23	0.23
20	0.29	0.29	0.29	0.29	0.29	0.29

^1^ The length for each window is 10 s. FFT was applied on data for each window.

## Data Availability

The datasets generated and analyzed during this work and the code developed in this work can be made available on reasonable request to the corresponding author.

## Abbreviations

The following abbreviations are used in this manuscript:

CSI	Channel state information
CW	Continuous wave
HVAC	Lighting, heating, ventilation, and air-conditioning
LO	Local oscillator
PIR	Passive infrared
RF	Radio frequency
RX	Receive
SoC	System-on-chip
TX	Transmit
WSSN	Wireless smart sensor network
